# A School-Based Mobile App Intervention for Enhancing Emotion Regulation in Children: Exploratory Trial

**DOI:** 10.2196/21837

**Published:** 2021-07-14

**Authors:** Bettina Moltrecht, Praveetha Patalay, Jessica Deighton, Julian Edbrooke-Childs

**Affiliations:** 1 Evidence-based Practice Unit University College London & Anna Freud National Centre London United Kingdom; 2 Department of Psychiatry University of Oxford Oxford United Kingdom; 3 MRC Unit for Lifelong Health and Ageing University College London London United Kingdom

**Keywords:** emotion regulation, digital mental health, mhealth, school intervention, child mental health, mobile phone

## Abstract

**Background:**

Most mental health disorders are first experienced in childhood. The rising rates of mental health difficulties in children highlight the need for innovative approaches to supporting children and preventing these difficulties. School-based digital interventions that address shared risk factors and symptoms, such as emotion dysregulation, present exciting opportunities to enhance mental health support for children on a larger scale.

**Objective:**

This study investigates the use of a new app-based intervention designed to support children’s emotion regulation in schools. The aim is to optimize the usability, acceptability, and utility of the app and explore its scope for implementation with the target user in the school context.

**Methods:**

As part of an interdisciplinary development framework, the app is being evaluated in a 3-month trial across 4 primary schools. In total, 144 children (aged 10-12 years) took part and accessed the intervention app in the classroom or at home. Outcomes regarding usability, acceptability, and implementation opportunities were assessed through digital user data, self-report questionnaires (132/144, 91.6%), and semistructured interviews with children (19/144, 13.2%) and teachers (6/8, 75%).

**Results:**

The app usage data showed that 30% (128/426) of the users were returning users. Self-report data indicated that 40.1% (53/132) of the children had not used the app, whereas 57.5% (76/132) had used it once or more. Of the children who had used the app, 67% (51/76) reported that the app was helpful. Interviews with children and teachers suggested positive experiences with the app and that it helped them to calm down and relax. Children reported that they perceived the app as acceptable, usable, and helpful. In terms of the intervention’s usability, most features functioned well; however, certain technical issues were reported, which may have led to reduced engagement levels. Teachers not only reported overall positive experiences but also discussed access difficulties and reported a lack of content as one of the main barriers to implementing the app. Having a web-based app significantly enhanced accessibility across devices and settings and provided teachers with more opportunities to use it. We identified the need for new, activating app features in addition to the existing, primarily relaxing ones. The findings indicated that it is possible to use and evaluate an app intervention in the school context and that the app could help enhance children’s emotion regulation. We discuss areas for improvement regarding the app, study design, and future implementation strategies.

**Conclusions:**

We share important insights with regard to the development, implementation, and evaluation of a new app for supporting children’s emotion regulation in schools. Our results demonstrate that mental health apps represent a promising means to facilitate effective mental health service provision in and outside of the school context. Important lessons learned are shared to support other researchers and clinicians on similar journeys.

## Introduction

### Background

Approximately 10%-20% of children and young people worldwide experience mental health problems, making it one of the leading causes of disability in this population [1-3]. Considering the significant impact of mental health problems on other developmental outcomes (eg, academic achievement and physical health) [4,5], human and economic costs are substantial, calling for new, innovative approaches to support children and to prevent this problem. To address this challenge, this study explores the use of a newly developed web-based app as a universal school intervention to support children’s mental health and well-being by strengthening their emotion regulation skills.

Schools have long been identified as an ideal setting to provide youth mental health support [6,7] because of the considerable amount of time children spend at school. Moreover, it has been suggested that implementing mental health interventions in schools can help overcome important social and environmental barriers to accessing mental health services, such as costs, family demographic factors, transport, and social stigma [8,9]. Evidence from 52 systematic reviews and meta-analyses has suggested that schools are key facilitators for the development and implementation of effective mental health interventions [10]. However, environment-specific challenges, such as increased pressure on teachers to meet academic targets, are pertinent to the implementation of new interventions in schools. Thus, interventions that require a significant amount of time and effort from teachers are often discarded [11]. Digital interventions provide an effective means to overcome such challenges, as they minimize costs, time, and personal resources in comparison with face-to-face interventions [11].

### Digital Mental Health Interventions for Children and Young People

Digital mental health interventions have received increasing attention in recent years [12], with growing evidence supporting their effectiveness in clinical and community settings [13,14]. Owing to the rapid and consistent progress of technology, children and young people are increasingly adopting mobile apps, and their usage should therefore be considered in today’s youth mental health provision. We built on these developments and designed a new web-based app to support children in the school context, which remains widely underexplored. To date, only a few app interventions have been developed and evaluated, specifically for children and young people [15,16], and even less so in the school setting, with some exceptions [17]. Early findings from this research indicated that school mental health apps were perceived as potentially useful. However, they also highlighted that future studies need to focus more on refining and adjusting digital interventions to meet young users’ needs and make them suitable for the context in which they are being delivered [18]. In line with this, it was highlighted that an iterative approach of testing, evaluating, and refining is integral to the development and design of digital interventions.

Newer evaluation guidelines for digital interventions consistently emphasize that any early evaluation attempts should focus on optimizing a digital intervention to ensure adequate levels of usability, acceptability, and engagement before any feasibility or efficacy testing [19,20]. Given that effectiveness trials are highly resource demanding, it is recommended that researchers follow a staged and iterative evaluation process [21], particularly with digital interventions. Digital interventions come with an added layer of complexity due to the underlying technology, which needs to be tested for its stability and usability first, as any issue with the technology would otherwise have a tremendous effect on the intervention’s implementation success and effectiveness as a whole [22,23]. Pursuant to this, the primary aim of this study is to optimize the existing app intervention by exploring its usability and acceptability from a user’s perspective as well as the possibility of implementing and evaluating it within the school context.

A closer look at the digital mental health landscape indicates that most interventions aim to support specific mental health disorders or symptoms [6,13,18]. However, especially in children where comorbidity rates (ie, transitioning between disorders and showing symptoms from more than one disorder) are high, it has been shown that transdiagnostic interventions are not only highly beneficial for this group but also that traditional psychological interventions can be further improved by adding a transdiagnostic focus [24]. Furthermore, transdiagnostic interventions have also been regarded as highly suitable for the school context, as they reduce the burden on teachers having to deliver multiple, highly fragmented, targeted interventions [25].

Evidence suggests that emotion regulation is an important transdiagnostic mechanism that underlies a wide range of mental health disorders and predicts later levels of psychopathology. Emotion regulation can be described as the extrinsic and intrinsic processes through which individuals monitor, evaluate, and modify emotional reactions to accomplish their goals [26]. There has been an increasing interest in emotion regulation as a treatment and prevention target because of growing evidence demonstrating that emotion regulation difficulties are not only present in most mental health disorders but are also a significant risk factor for future mental health difficulties [27,28]. This is supported by a number of systematic reviews [29] and meta-analyses [30,31], which have indicated that positive changes in emotion regulation are associated with a reduction in anxiety, depression, substance abuse, eating, and borderline personality disorder symptoms.

However, most digital mental health interventions have been developed for specific disorders, thereby leaving a significant gap in technologies addressing transdiagnostic factors such as emotion regulation. Although this could ultimately support a wider range of mental health problems, it is also highly suitable for the school context [32]. Most existing digital emotion regulation interventions have focused on emotion regulation deficits specific to either autism spectrum disorders or attention-deficit/hyperactivity disorder [33,34]. Although it has been shown that these interventions could potentially improve some of the specific deficits [35-37], the interventions were specifically designed for this unique population, thereby making it difficult to transfer them to a wider population. A very limited amount of research has explored the use of mobile apps to enhance emotion regulation in young people more generally [38] or as a means to prevent difficulties from arising. One exception is the new music app by Hides et al [38], which aims to enhance emotion regulation in adolescents and young adults. Their initial findings based on 169 young people (age=16-25) suggested that the app could potentially enhance emotion regulation in this population; however, further testing is required to determine its effectiveness.

To the best of our knowledge, there is currently no app intervention that targets emotion regulation in late childhood or preadolescence, despite an increasing number of scholars highlighting this period as a critical developmental stage to achieve maximum effect [39] in terms of youth mental health prevention. More specifically, past evidence has indicated that the median onset for most mental health disorders lies at the age of 11 years [40], with the majority reaching a peak during adolescence [41]. Recent developmental models suggest that adolescents are particularly prone to developing social-emotional disorders, which are triggered by a combination of developmental changes and preadolescent risk factors related to emotion regulation [39]. It has been argued that developmental changes hamper important emotion regulation processes, which subsequently lead to increased mental health difficulties later in adolescents [42]. Moreover, in the United Kingdom and many other countries, late childhood is marked by a transition period in which children have to leave primary school to enter secondary school. This period is frequently experienced as highly stressful by children [43], thereby supporting our case to strengthen children’s emotion regulation before this significant transition period.

Taken together, we believe that by addressing emotion regulation during childhood, our app intervention presents a promising means not only to prevent mental health difficulties from arising but also to serve as an early intervention tool for children who might already be experiencing such difficulties.

### This Study

There is a significant lack of digital mental health interventions for children that target important transdiagnostic factors, such as emotion regulation. The development of an acceptable, usable, and engaging emotion regulation app, which can be implemented successfully in the school context, will be highly beneficial for supporting children’s mental health on a larger scale. Therefore, this study explores and evaluates the use of a new emotion regulation app for children with the primary aim of optimizing it further and informing future development stages with the ultimate goal of making it highly suitable for the user group and context [18].

The following research questions are addressed:

1. How acceptable and usable is the app from the children’s perspective in the school context?
2. How do children interact and engage with the app at school?
3. What are the perceived barriers to and facilitators of implementing and delivering the intervention in the school context?
4. How can the existing app intervention be further improved?
5. What possibilities, facilitators, and barriers exist in terms of evaluating the app in the school setting?

## Methods

### Recruitment and Participants

The study was advertised on the Anna Freud National Centre for Children and Families organization’s website and in a newsletter that was sent out monthly to a network of schools and related organizations across the United Kingdom. Newsletter recipients had previously signed up to receive newsletters. Initially, 19 schools indicated an interest in participation. Of the 19 schools, 11 were primary schools and were invited to an initial phone call. During the initial phone call, we discussed the research project and intervention as well as the schools’ involvement if they agreed to take part in it. Only primary schools in the United Kingdom with access to tablets and wireless internet were eligible. Following the initial phone call, 4 schools were excluded: 3 were not primary schools and one had no phone or tablet policy. Three other schools stepped down before the start of the trial for the following reasons: (1) the research aspect of the intervention would take up too much time, (2) for a large percentage of parents, English was not their first language; hence, they struggled to understand the consent forms or information sheets, and (3) a lack of parental engagement.

Ultimately, 4 primary schools participated in this trial. Only children between the ages of 10 and 12 years with parental consent and child assent were eligible to participate, which resulted in data of 144 children at baseline and 132 children postintervention. Children’s ages ranged between 10 and 11 years (mean 10.5, SD 0.49). Of the total sample, 56.3% (81/144) indicated that they were White, 6.9% (10/144) were Black, 18.8% (27/144) were Asian, 15.3% (22/144) were mixed, and 2.8% (4/144) chose *other* as their ethnicity. Of the 144 children taking part, 166 (81.1%) children indicated that English was their first language, 25 (17.4%) children were not, and 2 (1.4%) children preferred not to provide that information. A total of 6 female teachers participated in postintervention interviews; 2 teachers from 1 school were not available because of their illness.

### Intervention

#### Intervention Development and Design Process

The app has been designed as a school-based universal intervention for children at the end of their primary school years (age: 10-12 years). The app is considered as a complex mental health intervention as it involves a set of multiple, interconnected, and interacting components [44,45]. As part of the development process, we created a new design and development framework based on the Medical Research Council (MRC) framework [44,46], the Patient-Clinician-Designer Framework [47], and the co-operative inquiry framework to co-design with children by Druin [48].

At this stage, we only focused on the first three phases of the MRC framework: theory, modeling, and exploratory trials. The theory stage concerns the exploration of relevant theory and a review of existing evidence to ensure that the most reliable intervention components are chosen. The intervention modeling stage suggests that the researchers focus on identifying potential underlying mechanisms that influence the preferred outcome, which are then included in the intervention. Following the design of the initial intervention, the researcher is advised to explore its components further through exploratory trials to identify constant and variable components, including acceptability of intervention, compliance, delivery, recruitment, and retention rate.

The MRC framework provides valuable guidelines for the development and evaluation of complex interventions; however, it provides little information with regard to the actual design of appropriate content [49]. Hence, we drew on two other frameworks rooted in the field of human-computer interaction and design. The Patient-Clinician-Designer Framework provides guidance on the design structure and content creation process of digital interventions for mental illness [47]. It aims to meet the complex requirements when designing user-centered interventions for mental illness by considering different perspectives (eg, in this study, teachers, parents, and children) and design goals.

As our main target group is children, we also drew on the co-operative inquiry framework by Druin [48], which provides specific techniques on involving young users in the design process of technologies. The co-operative inquiry framework highlights the importance of involving children as partners in the whole process, instead of merely letting them test the almost finished prototype or end product. Druin [48,50] also emphasizes on the benefits of conducting fieldwork (ie, *contextual inquiry*), especially when working with children, which allows researchers to detect relevant contextual information, including patterns of activities, ways of communication and other artifacts. In addition, it has been reported that discussing design features in the relevant context (eg, school or home) makes it easier for children to express ideas and provide suggestions.

**Figure 1:**
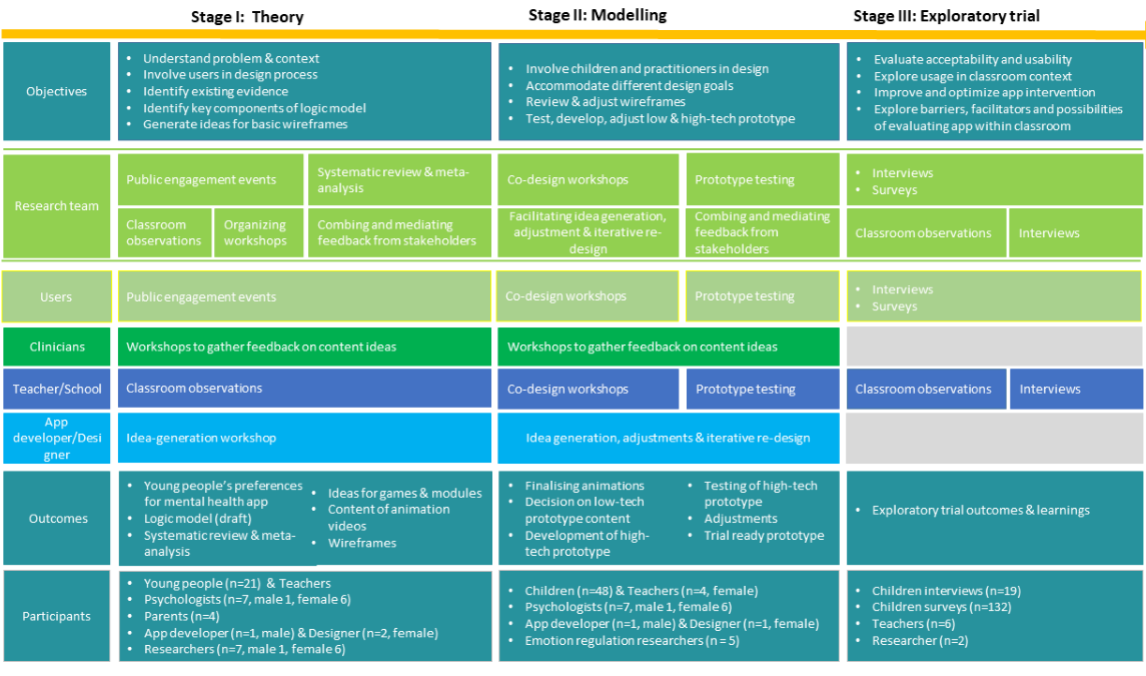
The 3-stage interdisciplinary development process, including research activities and stakeholders.

By combining the three frameworks from different fields, we hope to ensure a truly interdisciplinary approach to developing the app, the lack of which has been frequently criticized in many digital interventions [16,51]. Our development framework consisted of three stages, with each stage using a unique set of objectives, methodologies, and stakeholders (Figure 1). A more detailed description of the development and design process was published as a preprint by the first author (BM) and can be found in a study by Moltrecht et al [52].

#### Intervention Description

During the onboarding process, users were presented with a video that explains the purpose of the app and its usage. Next, the user was guided through a process to set up an account and then select a preferred color scheme and profile picture.

**Figure 2:**
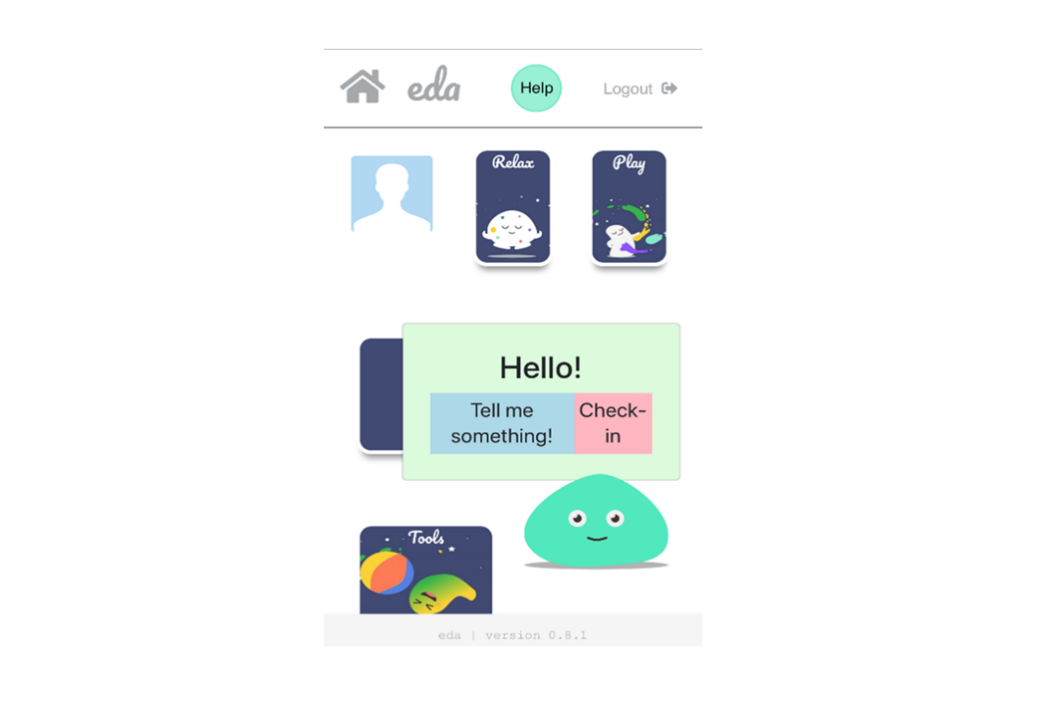
Home screen of the app with 4 main modules and the activated digital agent showing the “tell me something” and “check-in” function.

**Figure 3:**
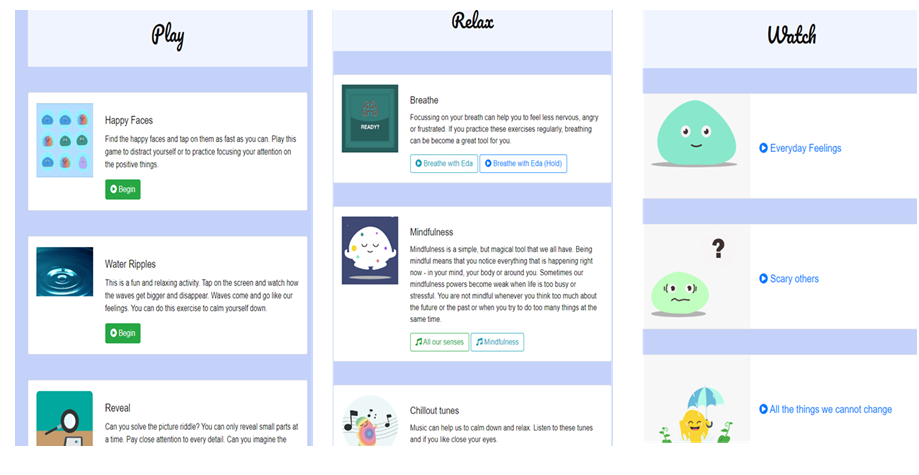
In-app content of the “play”, “relax”, and “watch” modules.

Once the user entered the home screen, they could choose from four modules: play (including four games), relax (includes mindfulness and relaxation exercises), watch (includes psychoeducational animations), and tools (includes a list of emotion regulation strategies), which provide users with the opportunity to learn, practice, and develop new emotion regulation skills (Figure 2, Figure 3). The content is presented through audio tracks, images, animated films, and games. A more detailed presentation of each module can be found in Multimedia Appendix 1 as well as the following preprint, which was published in a study by Moltrecht et al [52].

**Figure 4:**
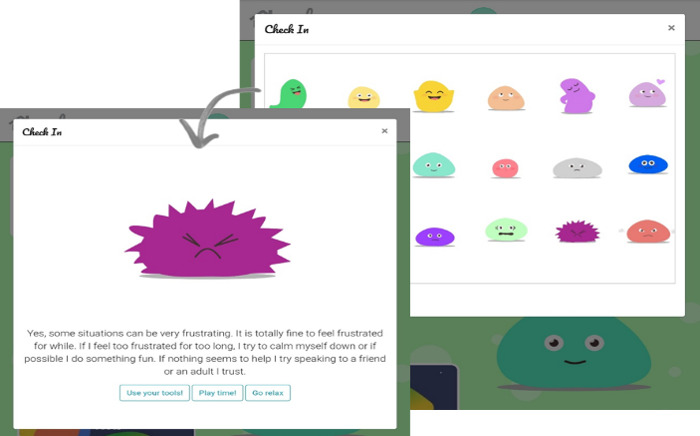
Check-in function in the app with "feeling frustrated" being selected.

An animated agent is located at the bottom-right corner of the home screen and opens two more features when the user taps on it. These features are (1) *tell me something*, which activates jokes and funny facts with the aim to increase levels of engagement; and (2) a *check-in* function, where users can select from a range of emotions about how they feel and are subsequently provided with more information about the particular feeling and suggestions for potentially helpful strategies (Figure 4).

Teachers and children were instructed to freely explore different ways of using the app intervention. By providing them with the link to the app, it was also possible for children to explore the app outside the school context if they wanted to. This flexible approach was adopted so that children and teachers could use the intervention in their preferred ways and hopefully perceive it as less of a burden. Furthermore, we expected this to increase our understanding about app usage and implementation in future trials.

#### Technical Specifications

The intervention was developed as a responsive web-based app, which was believed to increase the accessibility of the app, as it allowed users to access it across different mobile devices, as well as desktop computers and smartboards. Although it worked across multiple platforms, it was optimized for tablets, as young children are more likely to have access to tablets at school and at home [53].

The app is delivered through the browser, meaning over-the-wire updates can be pushed out instantly, and the app uses advanced HTML5, CSS3, and JavaScript (ES6) techniques to render a smooth and performant user experience. The underlying development platform used was Meteor.js, a full-stack Node.js application development framework, hosted on a resilient AWS EC2 (Amazon Elastic Compute Cloud) instance with a MongoDB database hosted via MongoDB Atlas. The app only requires an internet connection when users access it for the first time, after which it can be saved to the home screen of the device. This feature was chosen to mitigate the risk that the intervention could not be accessed when schools had reduced or limited Wi-Fi infrastructure. The app does not store any individual user data and adheres to the existing general data protection regulations.

### Measures

#### Demographics

Children reported age, gender, ethnicity, and their primary language spoken.

#### Internalizing and Externalizing Symptoms

The Short Mood and Feelings Questionnaire consists of 13 items that assess depressive symptoms in children and adolescents [54]. The Short Mood and Feelings Questionnaire has been shown to have good construct and internal validity across clinical (Cronbach α=.85) and community samples [55]. Furthermore, 5 items (“I get very angry,” “I lose my temper,” “I hit out when I am angry,” “I do things to hurt people,” and “I break things on purpose”) of the Me & My Feelings Questionnaire were added to assess externalizing symptoms [56]. The Me & My Feelings Questionnaire was developed as a self-reported mental health measure for the school setting and has been shown to have good psychometric properties across clinical and community samples (Cronbach α=.78-.82) [56,57]. Items on both scales were rated on a 3-point Likert scale, ranging from “Not true” (1), “Sometimes” (2), and “True” (3). With both scales combined, scores ranged between 18 and 54, with higher scores indicating greater levels of symptoms.

#### Well-being

The Satisfaction with Life Scale for Children [58] was used to assess participants’ personal perceptions of their well-being and satisfaction in life. The Satisfaction with Life Scale for Children consists of five items that are rated on a 5-point Likert scale, ranging from “disagree a lot” (1) to “agree a lot” (5) and has been reported to have good psychometric properties (Cronbach α=.84 [58]). Higher scores indicated greater levels of satisfaction with life and well-being.

#### Emotion Regulation

The How I feel - Questionnaire is a multidimensional self-report scale to assess emotional arousal and regulation abilities in children. It consists of 30 items rated on a 5-point Likert scale ranging from “very true of me” (5) to “not at all true of me” (1). The items assess the frequency, intensity, and regulation of five different emotions: sadness, fear, anger, happiness, and excitement. There are three subscales: (1) a positive emotion subscale, where higher scores indicate that happiness and excitement are experienced with high frequency and intensity; (2) a negative emotion subscale with high scores indicating that fear, anger, and sadness are experienced with high frequency and intensity; and (3) an emotion regulation subscale, where high scores reflect a strong ability to regulate the frequency and intensity of either positive or negative emotions.

The How I feel - Questionnaire has been reported to be a reliable and valid measure (Cronbach α=.84-.90) for research and for school interventions targeting pupils’ emotion regulation [59,60].

#### Engagement

Engagement data were collected through Google Analytics and paper questionnaires, in which children were asked how often they had been using the app in the past 3 months.

Teachers were provided with a logbook, which they were asked to complete on a weekly basis to describe how often and in what way they used the app in their classrooms. Items in the logbook included (1) what was the average time or preferred way to use the app, (2) how did children engage with the app (eg, tablet or smartphone), (3) how often was the app used due to classroom disruptions, and (4) average time to reinstate children into the class after app use?

#### Usability and Acceptability of App and Evaluation Measures

To increase our understanding of the usability and acceptability of the app intervention, we conducted brief semistructured interviews with teachers and children after the 3-month intervention phase. A detailed interview schedule can be found in Multimedia Appendix 2. The interview assessed (1) what children and teachers thought about the app; (2) whether it was easy to use; (3) what aspects of the app they found helpful or unhelpful; and (4) how they used the app, what aspects of it, and in what situations.

The research team also explored the usability of the evaluation methods used. The researchers were present when the children filled in the questionnaires to observe if they experienced any difficulties when completing them. Furthermore, the research team kept a logbook of significant events and conversations with teachers or any reported difficulties during the study.

### Study Design and Procedure

The University College London Research Ethics Committee approved this study (approval number: 7969/001).

Schools signed a memorandum of understanding, which explained the nature of the project and outlined the timeframes and responsibilities of the research team and the school. Parental consent, child assent forms, and parent and child information sheets were sent to the school and distributed by the class teacher. The research team visited the schools on the first day of the intervention to collect parent consent forms and child assent forms. Parents who indicated on the form that they had any remaining questions, where contacted by the research team to answer any remaining questions and obtain their oral consent over the phone, which was audio-recorded. Questionnaires were distributed to all participating children and the app was introduced to the class. After 6 weeks, the research team contacted the school to discuss the use of the app and any difficulties. Following a 3-month intervention phase, questionnaires were distributed again, and a researcher visited each classroom to observe the use of the app. Following this, semistructured interviews with 19 children and 6 teachers were conducted. Some teachers either had spoken to the children beforehand if they wanted to take part in the interviews or had asked the whole class in the presence of the researchers who would like to tell the researchers more about using the app at school. The research team highlighted to all children and teachers that honest answers were the most helpful and that they should not feel shy to report any negative experiences. All interviews with the children were audio-recorded with encrypted dictaphones and later transcribed. Owing to logistical issues, teacher interviews were not audio-recorded, and answers were written down by a researcher during the interview.

### Analytic Strategy

#### Quantitative Data

Quantitative data from the questionnaires were used to calculate descriptive statistics for the baseline and postintervention assessments using SPSS (IBM Corporation).

**Figure 5:**
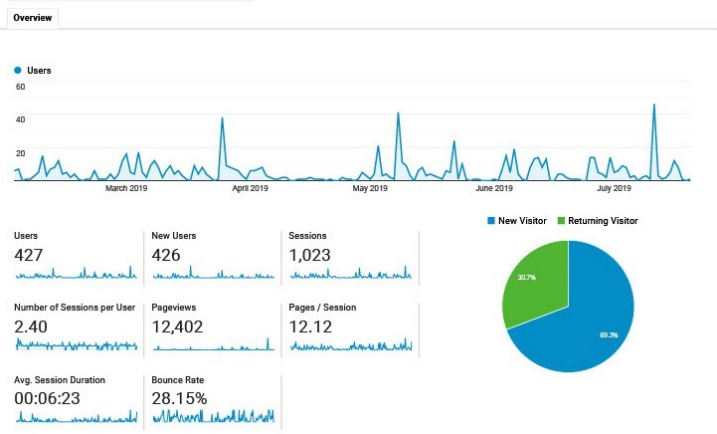
Engagement data as derived from Google Analytics.

Google Analytics data are presented below (Figure 5) to show overall usage and engagement.

#### Qualitative Data

The transcribed interviews and notes taken during the interviews were analyzed using thematic analysis [61].

Thematic analysis is a flexible method that can be used to analyze qualitative data by identifying patterns in the data. In this study, no existing framework was used but patterns were identified with the specific research questions regarding usability, acceptance, user-intervention interaction, and implementation in mind. Braun and Clarke [61] outlined 6 steps as a structured but flexible way to conduct thematic analysis (the article by Braun and Clarke [61] provides a detailed description of the six steps).

## Results

### Participants, Recruitment, and Retention

In total, 19 schools indicated an interest in taking part, of which we assessed 11 schools for their eligibility. Seven of these schools met our criteria and were eligible for participation. Of the seven eligible schools, 3 schools stepped down before the trial (see the reasons provided in the *Recruitment and Participants* section). In the final study, which included 4 primary schools, a 57% (4/7) retention rate was achieved.

In total, 144 children (female: n=79; male: n=62; not specified: n=3) completed the surveys at baseline and 132 children completed the surveys postintervention, thereby resulting in an attrition rate of 91.6% (132/144).

Six out of eight teachers, all female, participated in the postintervention interviews. None of the teachers completed the weekly usage logbook.

### Mental Health and Emotion Regulation Measures

The mean scores and SDs for pre- and postintervention assessments are presented in Table 1. Between 87.5% (126/144) and 91.6% (132/144) of the children completed the measures at baseline and follow-up. Although the mean scores and SDs are comparable with those found in other population and community samples [56,60,62], children in this study reported some difficulties with items on how they feel. Some children were unsure about the difference between items asking them about *strong* feelings and items asking them about *powerful* feelings.

**Table 1:** Descriptive statistics for mental health and emotion regulation questionnaires (N=144).

Outcome	Participants, n (%)	Score, mean (SD)
SMFQ^a^ baseline	144 (100)	25.09 (6)
SMFQ postintervention	132 (91.6)	24.22 (5.99)
SWLS-C^b^ baseline	140 (97.2)	4.13 (0.79)
SWLS-C postintervention	126 (87.5)	4.13 (0.83)
HIFQ-PES^c^ baseline	144 (100)	3.87 (0.84)
HIFQ-PES postintervention	132 (91.6)	3.72 (0.87)
HIFQ-NES^d^ baseline	144 (100)	2.11 (0.79)
HIFQ-NES postintervention	132 (91.6)	1.88 (0.72)
HIFQ-ERS^e^ baseline	144 (100)	3.37 (0.83)
HIFQ-ERS postintervention	132 (91.6)	3.35 (0.87)

^a^SMFQ: Short Mood and Feelings Questionnaire. Lower scores indicate fewer internalizing and externalizing difficulties.

^b^SWLS-C: Satisfaction With Life Scale. Greater scores (maximum total score=5) indicate greater satisfaction with life.

^c^HIFQ-PES: How I feel - Questionnaire–Positive Emotion Scale. Greater scores indicate a greater frequency and intensity of happiness and excitement.

^d^HIFQ-NES: How I feel - Questionnaire–Negative Emotion Scale. Greater scores indicate a greater frequency and intensity of sadness, anger, and anxiety.

^e^HIFQ-ERS: How I feel - Questionnaire–Emotion Regulation Scale. Greater scores indicate the greater regulation of the frequency and intensity of emotions.

### Engagement

In total, 57.5% (76/132) of all children from all schools indicated that they had used the app at least once, whereas 40.1% (53/132) indicated that they had never used the app in the past 3 months. At postassessment, 7.6% (10/132) of the children reported that they had used the app weekly. Of the 76 children who indicated that they had used the app, 67% (51/76) said that they found it helpful, and 58% (44/76) said that they would recommend the app to a friend.

No data were gathered through the teacher logbooks; therefore, we were unable to analyze any quantitative data on average usage times per classroom over the 12 weeks or in what way the app was engaged with over time.

Data collected via Google Analytics indicated that 426 users had accessed the website, of which 30% (128/426) were returning users and 70.2% (299/426) were new visitors. Furthermore, the average time spent on the app per session was 6 minutes and 22 seconds and the *play* module was most frequently visited, followed by *relax* and *watch*.

### Qualitative Outcomes

#### Children’s Reported Usability and Acceptability

In total, 19 children shared their experiences using the app during the interviews. Most children reported positive experiences with the app and provided insights regarding specific strengths and weaknesses.

#### Feeling Calm and Relaxed

Nearly all children reported that using the app made them feel calm and relaxed. They indicated using the app, especially during stressful times (eg, *test at school, argument with friend or sibling, or having a bad day at school*). Some children reported using the app to fall asleep at night. Children seemed to enjoy two features in particular, the *Water Ripples* game and the *music* function:

I think the app’s helpful, um, if you’re stressed or if you like, it’s a good way to relax and you can use it to calm down.

#### Helpfulness

Most children reported that they found some of the features (ie, *check-in* function, videos, and tools list) particularly helpful and useful:

...the thing I also found was quite helpful was where you could sort of tell how you were feeling, and it sorta gave what you should do.

They explained that it increased their understanding or knowledge of their feelings and provided suggestions regarding possible solutions or actions to take:

...like it makes you understand it, your feelings are something in you and it’s ok to have them.

#### Design and Technical Issues

With respect to the app’s limitations, the children most frequently reported technical or design issues. Most commonly mentioned were problems (eg, “didn’t work,” “too slow,” or “took too long to load”) related to the *Reveal* game and video clips, which appeared to happen more frequently on some devices than others (this was assumingly related to certain web-browsers). Furthermore, children mentioned that they would prefer to have more options to choose from for the color scheme and design of the home screen and other personalization features.

#### Feelings of Anger

A few children reported that they found the app to be less helpful when they were very emotional or experiencing strong feelings of anger:

I liked the app. Although it did not help me with my anger, no one could help me with this yet.

In relation to this, one child explained that when they were angry, they preferred to “do something to kind of get it out” instead of engaging in calming or relaxing exercises.

#### Children’s Interaction With the Intervention

Children reported different preferences for where, when, and how to use the app.

#### Location

Most children used the app at school where it was introduced to them. Some children reported using the app primarily during times when teachers allowed them to choose an activity for a certain amount of time. Other children said that they had agreed with their teacher to use it in certain situations when they struggled to concentrate or participate in class. Almost half of the children (9/19, 47%) reported that they also used the app at home.

#### Emotional Prompts

Many children suggested that the app is most suitable during stressful times or when someone struggles with their feelings. They provided examples, including having a fight with someone, not being able to concentrate, or feeling bored:

The best way to use the app is if you’re stressed out, or um, if you need something to take your mind off something.

Children reported less frequent use of the app during less stressful times and when they felt generally happy:

It’s not for someone who is happy, but some people get a bit angry sometimes, I'd recommend it to them.

#### Design Prompts

Although most children reported that the app was easy to use (“I just knew how to use it”), it became apparent that some features, such as the help function, had not been accessed or had not been discovered by them. Furthermore, children reported that they had forgotten about the app when they had not used it for a while.

#### Access Barriers

As the app was web-based, many children were not able to find or download the app through the app store, which they reported was their primary way to access apps. Hence, this was one of the major barriers to accessing the app. One child said, “I couldn't remember what it was called, so I couldn’t find it.”

Furthermore, in relation to the school setting, children reported difficulties accessing tablets as they were either not permanently available or locked in a drawer, so they had to ask for it. The latter was also perceived as a barrier, as children were too shy to request it and “didn’t want to ask the teacher for it.”

### Teacher-Reported Facilitators to and Barriers of Implementation and Delivery

#### Access Barriers

Teachers also mentioned that the app was hard to find if the link was not available or in reach, which seemed to inhibit the use of the app from the teacher’s perspective.

#### Technical Issues

Teachers reported experiencing technical issues of video crashing or content taking too long to load, which was perceived as a barrier to using it.

#### Flexible Use

Every teacher reported a slightly different way of using the app in their classroom, with some preferring a whole-class approach and others directing individuals to the app. The freedom to use the intervention in different ways was perceived as a facilitator, as it made it easier for them to find opportunities to use the app in the classroom.

#### Compatibility With Teaching Style

Teachers were more likely to use the app if they were compatible with their existing teaching methods and did not require additional work or adjustments. Moreover, teachers liked that they could direct children individually to the app, when needed, and that it “doesn’t take away too much time from the teaching” or interrupts the classroom atmosphere.

Furthermore, in classrooms where mindfulness and relaxation exercises were already used in other ways, teachers reported to primarily use the relaxation module by projecting the exercises on the smartboard or playing the music, which seemed to “[help] to calm them [the children] down during work times.”

In relation to this, teachers provided further app suggestions to support their teaching (eg, a timer and noise meter with a traffic light system to signal children when they are too noisy) and could therefore further facilitate the implementation of the app.

Teachers who did not see suitable opportunities to integrate the app in their teaching reported that it took them some time to “remember the app” and that there was a tendency to rely on “old habits” or methods in difficult situations (“in the heat of the moment”). They also reported feeling confident that using the app could become a habit.

### Recommendations for Further Improvements

#### Content and Functionality

Both children and teachers reported that they would like more content. Some children reported that they “got a bit bored” by having to play the same game. Others mentioned that there were “only four videos” to watch, which resulted in decreased interaction with the app over time. In line with this, teachers indicated that it was more likely that they continued using the app if there were updates that were more frequent, including new content.

Furthermore, children reported a wish for more features, such as making the embodied agent more responsive, so that more interactions are possible. One child compared it with “a robot that you can talk to.”

#### Individual Needs

Some teachers mentioned that *activating* features, would be helpful “for children with too much energy,” “when they are angry,” or after “a long time of sitting.” One teacher suggested the use of dance videos as an activating exercise during breaks.

In addition, teachers saw a need for more interventions that specifically support children with learning disabilities or autism spectrum disorder, as they seem to be more likely to experience specific emotion regulation difficulties associated with the disorder.

## Discussion

### Acceptability and Usability of the App

Through questionnaires and postintervention interviews, the present study collected data on the perceived usability and acceptability of the app. The interviews suggest that children perceived the app as acceptable, usable, and helpful. The interviews provided preliminary evidence that the app helped children to calm down and relax in stressful situations, and potentially increased their understanding and knowledge of emotions. However, this needs to be thoroughly tested in future studies.

Some children reported that they found the app to be less helpful when they experienced anger. This suggests that there is a need for different types of support with respect to different emotional experiences. A similar idea was suggested in past research with infants, whereby certain strategies (ie, distraction) were more effective in regulating anger than fear [63].

In terms of the intervention’s usability, most parts of the intervention functioned well; however, certain technical issues were reported that led to reduced engagement levels. These issues must be addressed before future evaluations.

### Interaction and Engagement

Children reported that they used the app primarily at school, whereas others accessed it at home. Most indicated that stressful situations were one of the main motives for accessing the app. Children and teachers did not receive specific instructions on how to use the app; therefore, differences occurred between classes, with some teachers directing certain children to the app in a special area in the classroom, whereas others used it primarily with the whole classroom. Children were provided with a link to the app at school and they could access it at home if they followed the same link. Although this made it more difficult to exactly track usage, the open approach helped us understand how and when children used the app depending on the context (eg, listening to music to concentrate in class vs listening to music to fall asleep at home).

Engagement data from Google Analytics suggested that 30% (128/426) to 37.1% (158/426) of the users repeatedly accessed the app over a 3-month period. Although this number would ideally be higher, it is similar to adherence rates reported for other mental health apps [64].

In an attempt to mitigate low levels of engagement, one of the most common limitations in digital health interventions, we involved children and young people throughout the development and design of the app intervention. Interviews with the children indicated that the *Water Ripples* game was perceived as one of the most positive and helpful features in the app, which supports our interdisciplinary development approach.

With respect to children’s and teachers’ requests for having more content updates to maintain the level of novelty, future research could explore the use of timed updates, whereby sections of the content are released one after another. We recognize that this was only one piece of the puzzle. Issues surrounding user engagement are a recurring topic in the field, posing the question of how much engagement is actually needed for an intervention to be effective. The data from this study do not provide sufficient evidence to determine this, and further research is required.

Nevertheless, based on the children’s reports, it can be assumed that some of the features positively influenced user engagement, such as the digital agent, which should be further explored (eg, chatbots and getting clothes or objects as rewards to change its appearance).

### Delivery and Implementation

Teachers play a significant role in the intervention’s implementation and delivery. Therefore, we tried to gain insights into potential barriers to and facilitators for implementing the app in a school setting.

Although teachers reported positive experiences, they also reported access difficulties and lack of content as the main barriers to implementing the intervention. Our findings suggest that teachers are more likely to use the app if they are compatible with their existing teaching methods. Teachers who saw fewer natural opportunities to integrate it reported more barriers to use the app. This is in line with previous research findings [18] and highlights the importance of considering teachers’ perspectives when agreeing on design goals and intervention features. At the same time, teachers’ requests and any subsequent design implications will have to be carefully considered in relation to the intervention’s overall goal (ie, supporting children’s emotion regulation) and any conflicting interests with young users’ requests need to be mediated. For instance, although some teachers suggested features such as a noise meter, the research team needs to explore whether such a feature would primarily benefit teachers (eg, as a classroom management strategy) or young users as well.

Another barrier related to the school environment was the small number of tablets available per class, which limited the accessibility of the intervention. In addition, some schools only provided access to tablets upon request, thereby limiting ease of access. However, the possibility of accessing the app through other devices, such as computers or smartboards, enhanced the general uptake of the intervention in the school context.

### Possibilities, Facilitators to, and Barriers of Conducting a School-Based App Evaluation

Important conclusions can be drawn regarding the possibility of evaluating the app in a school setting. In terms of school recruitment, we retained 57% (4/7) of the originally recruited schools, which could be further improved. Schools reported that they feared that their resources were limited to facilitate the research. Furthermore, some schools reported that there were significant issues due to unmet translation needs, which suggests that translated information sheets and consent forms should be made available in a future trial. Some schools also mentioned that it would have been helpful if the research team had provided guidance that was more specific on the app usage or if the research team had explored different means to use the app with the teachers beforehand.

In terms of the measures used, a high number of questionnaires were completed at baseline and follow-up (baseline: 126/144, 87.5%; follow-up: 132/144, 91.6%), thereby suggesting acceptable completion rates. However, none of the teachers completed the weekly logbooks, and it was not possible to record any of the interviews with the teachers, which highlights specific barriers in terms of data collection from teachers.

Despite the identified barriers, the findings suggest that it is possible to implement and evaluate the app in a school setting. However, we suggest that a comprehensive feasibility trial is conducted next to ensure (1) an enhanced recruitment and assessment strategy, (2) improved integration of the app intervention in the school curriculum and teaching methods, and (3) easier access to the app by making it available on the app store. In line with this, we also suggest that a set of feasibility criteria is defined beforehand, so that informed decisions can be made as to whether an effectiveness trial is the next appropriate step [65].

### Considerations for Future App Features and Research

In addition to the suggestions above, we would like to share further lessons learned, which will hopefully help improve the present and similar school-based app interventions.

### The School Setting

With respect to one of the primary design goals and the prioritization of engagement, we opted for a multimedia app that included various audio and video materials. However, this partly presented itself as unsuitable for the school environment, as sounds can be disturbing or require access to headphones. This observation emphasizes that new types of interventions are accompanied by new challenges, which need to be explored further and taken into account.

### Specific Emotion Regulation App Features

On the basis of previous research, we included a range of mindfulness and relaxation exercises [66], which children experienced as positive. Although mindfulness and relaxation apps have gained increased popularity, this study shows that for certain negative emotions, *activating* features instead of calming features could be equally important. These features seem to tap into a different set of emotion regulation strategies (eg, behavior activation and physical activity), which should not be neglected [67,68]. The finding that some children needed something else when they were angry is in line with emotion regulation research indicates that effective emotion regulation not only is merely about the frequency by which we apply certain strategies [69,70] but also is about whether we have access to a diverse set of emotion regulation strategies that can be flexibly applied depending on the situation’s demands [71,72]. This highlights the complexity of the emotion regulation construct, which is sometimes applied in an overly simplistic manner [73,74]. To account for this complexity, we chose to develop psychoeducational films to introduce children to the more complex concept of emotion regulation. Interviews showed that films could have the potential to increase children’s understanding and knowledge of emotion regulation, but more research is needed to prove this.

Another important feature to enhance emotion regulation was the *check-in* function, which was designed to support children by accessing adaptive emotion regulation strategies and increasing their awareness of the diverse range of emotions. Research has shown that both are related to positive mental health outcomes [75-77]. Children in this study found this feature helpful and used it either as a guide to identify their feelings or to explore and learn about different strategies. We believe that this feature could also serve as an ideal assessment tool for collecting data about users’ day-to-day feelings.

Concerning the *help* function, we found that many children had not used or seen it until a researcher mentioned it in the interview. After consulting the children, most agreed that it was a good idea to provide guided support for emotionally intense situations. However, they also explained that having it in the app did not address their needs in the situation, due to a lack of permanent or immediate access to a device. We suggest that future research could explore this idea further through wearable devices, which also provide an ideal opportunity to integrate advanced health technology concepts, such as just-in-time adaptive interventions [78].

### Strengths and Limitations

A significant strength of this research concerns the development process of the app, for which we included children and young people at every stage and adopted a truly interdisciplinary approach [52]. Another strength relates to the collaborative approach with schools, which has various benefits. It ensured regular access to the user group and helped identify context-specific design goals at an early stage. Teachers also contributed tremendously to their views and expertise from working with the user group. The lack of teachers’ roles as intervention deliverers is a limitation that needs to be addressed further. Furthermore, teachers had very limited time available; therefore, we suggest conducting more regular classroom observations to adjust the app for the classroom setting.

Finally, we would like to mention that in some cases, teachers played a significant role in selecting children for the interviews. Although we asked teachers to provide us with a representative sample, it is possible that teachers unconsciously selected children who were more likely to report positive aspects. This assumption is based on previous research showing that children with certain characteristics, such as lower academic achievement or greater externalizing tendencies, were less likely to be considered for taking part in research [79].

### Conclusions

We explored the possibilities of using, implementing, and evaluating a new app intervention to improve children’s emotion regulation abilities in the school context. The results suggest that the intervention presents a promising opportunity to enhance emotion regulation abilities by considering the complex nature of the construct itself. The app aims to assist children with their emotion regulation abilities by offering guidance in identifying feelings and selecting adaptive emotion regulation strategies.

The app was perceived as acceptable and usable, although some technological issues need to be addressed before any further evaluation. The data provided valuable insights regarding important facilitators and barriers to implementing and evaluating the app in the school setting. Important *lessons learned* are shared and will hopefully be beneficial in the development and evaluation of similar interventions. We hope that this work will motivate the development of further technology-based interventions that target transdiagnostic mechanisms such as emotion regulation in youth, as these are of particular importance considering the high comorbidity rates and less specific symptom profiles in children and young people.

## Appendix

Multimedia Appendix 1 Animation video showing an overview of app features and usage.

Multimedia Appendix 2 Interview script.

## Abbreviations

AWS EC2: Amazon Elastic Compute Cloud
MRC: Medical Research Council
