# Supplementary material for: A School-Based Mobile App Intervention for Enhancing Emotion Regulation in Children: Exploratory Trial
Source: JMIR Mhealth Uhealth. 2021 Jul 14;9(7):e21837. doi: 10.2196/21837 (PMC8319776; doi:10.2196/21837)
Supplement: Multimedia Appendix 2 [file mhealth_v9i7e21837_app2.docx]

**EDA Young Person Interview Schedule**

**Things to remember:**

- No interview can begin without a signed parental consent form
- Be flexible with interview or focus group format, depends what YP wants
- Make sure to narrate when showing YP EDA or when YP is drawing responses
- Prizing detail of response not praising responses
- Mention note-taking at beginning of interview
- “I don’t know”= could mean that they don’t understand question, try rephrasing, or if repeated could mean that they don’t want to answer, try checking in about whether they want to carry on with the interview

**INTRODUCTION:**

“Thank you so much for doing this interview with me. I have a few questions I’d like to ask about your experiences using EDA, there are no right or wrong answers I’m just interested in what you think. If you don’t want to answer a question or if it’s unclear then just tell me and we can skip it, or I can explain it. We will write up what we find from all of our interviews with young people your age using the EDA app and if you would like a copy of our findings please let your teacher know. Everything that we talk about today is private and confidential unless I’m worried that any harm is going to come to you or to anyone else. In which case I would need to speak to your teacher and my supervisor, whose name is Julian, but I would tell you if I was worried in this way first. You are welcome to stop the interview at any time”

**ICE BREAKER**

Tell me about pet/favourite football player/last weekend/your class

**QUESTIONS**

- What do you think about the EDA app?
  - Interesting?
  - Easy/difficult to use?
- What is your favourite thing to do in the Eda app?
- What is it that you like about this?
- Can you tell me about a time you used this part of the app?
- What part of Eda do you find most helpful?
  - What is it about this part that helps you?
  - How has it helped you? [Feelings or changed behaviour]
- Can you tell me about a time when it has helped you?
- What would you have done if you did not have the app to help?
- What parts of the EDA app do you not like/find unhelpful?
  - - How would they change this section/like this section to be different?
    - Is there anything teachers could be doing to help?
- How often have you used the EDA app in school?
  - Didn’t use it, one or twice, weekly, daily?
  - Tell me about a time when you used the app in school?
  - Do you know why you and your class used it or didn’t use it?
- How often have you used the EDA app at home?
  - - Tell me about a time when you used the app in school? What happened?
- How do you feel when you are using the EDA app?
- Can you think of situations in which you would like to access EDA outside of school? i.e. at home, on weekends, while doing your homework, spending time with friends
  - - What would be different if you had EDA in these situations?
- Have you ever noticed times where you thought your friends should know about the EDA app?
- Do you think it would help your friends? – If so why or why not?
- Would you recommend EDA to your friends?
- Would you recommend EDA to your family?

**CLOSING QUESTIONS:**

“Thank you very much again for doing this interview with me today; it’s been so helpful to speak to you. Do you have any questions for me now that we’ve finished the interview? We are going to use these interviews to help us understand how EDA helps young people. Would you like to choose a pseudonym for when we write up our findings? This is another name that we will use for you in our write-up to help ensure that other people don’t recognise you”

**END INTERVIEW**
